# Cost-analysis of different management policies for patients with mild hepatitis A virus infection in Kazakhstan

**DOI:** 10.1186/1478-7547-3-4

**Published:** 2005-05-12

**Authors:** Abdiaziz S Yassin, Michael Favorov, Edmond Maes, Ramses Sadek, Aliya Jumagulova, Victor Merker, Tatiana Surdina, Terence Chorba

**Affiliations:** 1 Division of International Health, Centers for Disease Control and Prevention (CDC), Atlanta, Georgia, USA; 2 CDC Central Asian Regional Office, Almaty, Republic of Kazakhstan; 3 Division of HIV/AIDS Prevention, CDC, Atlanta, Georgia, USA; 4 Almaty City Sanitary and Epidemiological Stations, Ministry of Health, Almaty, Republic of Kazakhstan

## Abstract

**Objective:**

For patients with mild hepatitis A virus (HAV) infection, this study compared estimates of total costs associated with managing cases under a policy of mandatory hospitalization in the Republic of Kazakhstan and estimates of total costs associated with managing cases in outpatient settings. Costs were estimated both from the perspective of the Ministry of Health and from a broader societal perspective.

**Methods:**

Data were collected by using a standardized structured questionnaire. For cases of mild HAV infection, medical records were obtained from 200 patients managed by hospitalization and from 251 patients managed in an outpatient setting. Personal interviews were also conducted to collect information on productivity losses and out-of-pocket expenses.

**Results:**

Nationally, we estimated about 21,600 cases of mild HAV infection annually. The mean annual treatment costs  in hospital for mild HAV infection was estimated at US$3.39 million (2001 US$) (95% confidence interval [CI] = [US$3.26 million – US$3.52 million]). The total annual mild HAV infection cost to the society, including direct medical and nonmedical costs and productivity losses due to 721,440 lost work days, was estimated at US$6.26 million (95% CI [US$6.05 million – US$6.47 million]). In sensitivity analyses, the total annual cost of mild HAV infection ranged from US$4.37 million to US$24.66 million. The survey results showed that a relatively minor change in the current policy of mandatory hospitalization could result in an estimated total annual savings of US$4.62 million (2001 US$) in Kazakhstan.

**Conclusion:**

Adoption of an outpatient management policy for cases of mild HAV infection would generate substantial cost savings to the Ministry of Health and society.

## Background

The average annual incidence rate of hepatitis A virus (HAV) infection varies significantly worldwide ranging from 9.1 per 100,000 in the United States and Europe to 500 per 100,000 in the Central Asian Republics [1,2]. According to the latest available data, the incidence of HAV infection remains as high as 150 cases per 100,000 population in the Central Asian Republics and Kazakhstan in 2001 [3,4]. Although HAV infection incidence has decreased slightly compared to the figures for the last decade, the persistence of high incidence in Kazakhstan is mainly due to problems related to poor supplies of good-quality drinking water, sanitation and hygiene practices [1,5,6]. It has been documented that the incidence of HAV infection is closely linked to poor socio-economic living conditions [3].

HAV infection is usually a mild illness, but can be severe if it occurs concurrently with other diseases or comorbid conditions [7-10]. In Britain, and North America, a patient with HAV infection is hospitalized only if (a) (s)he is too sick with diarrhea, nausea, and vomiting to be managed appropriately with antiemetics and oral rehydration at home, (b) (s)he has hepatic and/or renal failure (sometimes seen with HAV-infection in persons with underlying alcoholic liver disease or with chronic hepatitis caused by other viral agents) [11], or (c) (s)he has a clinical presentation that is truly anomalous (*e.g*., meningoencephalitis has been reported in association with HAV infection but is exceedingly rare [12]). However, in many republics of the former Soviet Union, HAV infection has been a cause for routine hospitalization, as mandated in administrative directives. The premise on which many such patients are hospitalized should be reconsidered because relatively few patients with HAV infection become seriously ill, no treatment to influence the underlying course of the disease exists, and neither admission to hospital nor isolation from others in the home lessens the spread of the infection [13].

Because HAV infection is neither a severe illness nor one with important sequelae, it has been observed that the rates of infection of family members of patients with mild HAV managed at home or in hospital were similar [13-15]. Previous studies using data from the region reported that the hospitalization of patients after the appearance of jaundice was unjustified, and that the quality of treatment between inpatient and outpatient settings would be similar [13,14]. However, in severe cases (<10% of HAV infections), patients are more likely to be managed in a hospital and to receive more intensive treatment, including ultrasound and computerized tomography exams, as well as liver biopsies [7,8,10]. In such cases, the assumption of similar quality of inpatient and outpatient treatment does not hold.

Using data gathered from patients with mild HAV infection in Almaty, Kazakhstan, we conducted a cost-comparison analysis of the policy of mandatory hospitalization and an alternative policy of outpatient management for patients with mild HAV infection. The purposes of this study were (a) to determine the mean treatment costs associated with managing cases of mild HAV infection in an inpatient setting under a policy of mandatory hospitalization, (b) to compare those costs with costs of treating patients with mild HAV infection using outpatient management, assuming a similar quality of treatment under inpatient and outpatient conditions, and (c) to determine total direct medical and nonmedical costs as well as productivity losses associated with mild HAV infection and then compare these costs both from the perspective of the resource-allocating authority (*i.e*., the Ministry of Health) and from a broader societal perspective.

## Materials and methods

### Data sources

Data were collected using survey questionnaires that involved interviews with patients and medical practitioners between November 2000 and February 2001. Information was collected on patient's age, sex, principal diagnosis, duration of illness, use of services, and cost data. Patients were interviewed to collect information regarding out-of-pocket and direct nonmedical expenses, such as expenses paid for transportation to outpatient clinics and indirect costs associated with the number of days absent from work. Interviews with medical personnel and hospital administrators were conducted to determine the costs and quantify the amount of health and nonhealth staff time spent per day in caring for patients with mild HAV infection.

A sample size of 451 subjects (200 inpatients and 251 outpatients) was used to conduct a cost-comparison analysis. We enrolled patients presenting to either of two hospitals in Almaty, Kazakhstan, in a geographical area where mandatory hospitalization for mild HAV infection was still practiced. We also enrolled patients in 19 outpatient clinics in geographical areas where mandatory hospitalization was no longer practiced. Patients selected for the study included members of both sexes, all age groups, and varied in ethnicity.

### Case definition

A case of mild HAV infection was defined as a clinical presentation with manifestations of acute hepatitis and serologic confirmation of HAV infection in a person who reported to one of the selected hospitals or outpatient clinic sites. The clinical manifestations of HAV infection included sudden onset of fever, anorexia, fatigue, and/or abdominal discomfort followed within a few days by jaundice and hepatomegaly [16-18]. Serologic confirmation of mild HAV infection was determined by elevated alanine serum transferase (AST) not in excess of 5 times the upper limit of normal of the reference value, and by presence of detectable IgM anti-HAV

Criteria for inclusion in this analysis were (a) having mild HAV infection as a primary or sole diagnosis confirmed by laboratory; (b) being between 1 and 65 years of age and being either hospitalized or managed at one of the study sites by a health care worker during the study period; and (c) providing voluntarily signed consent that included permission to review medical records. Persons who had multiple concurrent diseases or comorbid conditions and those who had not been diagnosed and tested positive for mild HAV infection more than 30 days before the study period were excluded from the study.

### Cost description

Costs for direct medical and non-medical services provided for patients with mild HAV infection were calculated based on interviews with medical providers, hospital administrators, health officials and patients. Units of direct medical services recorded included the number of days spent in hospital, health staff time allocated to treatment, and costs for those services specifically identified as part of the management of patients with mild HAV infection in hospital and outpatient clinics. Time spent by health care personnel was obtained based on reviews of medical records. Costs were based on estimates of costs for time spent by hospital- or clinic-personnel per day in caring for patients with mild HAV infection: in hospital, physicians spent approximately 54 minutes (min.), nurses approximately 89 min., and nonhealth staff about 40 min.; in the outpatient clinics, physicians spent approximately 26.4 min., nurses approximately 18 min., and nonhealth staff a negligible amount of time. In this analysis, we are considering only the base salary for the health care personnel; physicians are paid US$15.84 per 8-hour workday, US$4.8 per day for nurses, and US$2.4 per day for nonhealth staff obtained from the budget of each health facility and based on the national contract for health personnel. Although physicians earn more than their base salary through various bonus payments and through informal payments from patients, consistent estimates of these payments could not be captured and were not included in this analysis [6,19,20]. Since an estimated 80% to 90% of physician income is obtained informally [20], it is difficult to track the flow of incomes for doctors, and we were able to use only the base salary for our estimates.

The cost per patient-hospital day included the costs of purchase and repair of medical equipment and buildings, administrative and operational costs in the hospitals allocated to the treatment of mild HAV patients. To cover the full capital and recurrent cost, cost per patient-hospital day was determined based on average national estimates provided by hospital administrators [21-23]. Although hospital costs cover capital and recurrent costs such as the cost of building and medical equipment, laboratory services, health and nonhealth personnel, and hospital administrative costs, we estimated separately the costs for laboratory and personnel services for managing patients in hospital based on medical records and interviews with medical practitioners. Costs for hospital meals and prescription drugs were obtained based on patient self-reported out-of-pocket expenses. The cost of hospital meals was estimated for the respective number of inpatient hospital days. The number of hospital days was determined based on patient medical records. Because all patients paid for hospital meals and prescription drug charges where incurred, we calculated the cost of hospital meals and prescription drugs separately.

Total treatment costs were calculated as the sum of direct medical and non-medical costs which included hospital costs, costs for time spent with patients by physicians, nurses, and other staff, laboratory and prescription drug costs, as well as non-medical costs such as hospital meals and transport. Because there are no long-term sequelae of mild HAV infection [7,10], no home care was needed for such patients; therefore, the cost of home care was not calculated. Productivity losses were calculated as indirect costs associated with lost workdays due to illnesses of HAV infection. The number of lost workdays were determined based on self-reports of missed work. The exchange rate used was US$1 = 148 Kazakhstan Tenge (KZT), a rate that has not varied more than 15% since January 2001. As of May 5, 2005, the exchange rate was US$1 = 131 KZT.

### Treatment outcome

Having measured the direct medical and nonmedical costs, as well as productivity losses of managing mild HAV infection, we defined effective treatment or management for a mild HAV infection as an episode of care (which included all services provided) that led to a complete recovery. As has been observed epidemiologically elsewhere, we assumed that the rates of infection of family members of patients with mild HAV infection managed at home or in hospital were similar [13,15].

### Statistical analyses

We completed our analyses in five stages: First, we estimated the mean annual treatment cost per case of mild HAV infection when managed in inpatient and outpatient settings. Using SAS statistical programs, comparison between inpatient and outpatient groups were made using PROC TTEST of the null hypothesis of equal group variances, and we determined the difference in mean treatment cost per case using t tests of differences in means. The confidence interval (CI) for the difference between two sample means was obtained using a "pooled" estimate of the standard deviation [24].

Second, we stratified the samples by four different age groups, given significant differences in mean age between the two groups. We categorized patients into four age groups: ≤ 18, 19 – 26, 27 – 34, and ≥ 35 years to estimate the mean annual treatment cost per case of mild HAV infection by age group. We used PROC GLM to conduct one-way analysis of variance (ANOVA) using Bonferroni t tests of pair of means within age groups to test the null hypothesis of equality of the group means among different age groups.

Third, we estimated the number of lost workdays for both inpatient and outpatient group. We calculated the productivity losses by multiplying the number of lost workdays by employed patients aged 18 – 64 years or by adult parents or guardians of children with mild HAV infection, and by daily urban wage rates. In this analysis, we did not consider replacement costs for unemployed patients.

Fourth, we estimated the total annual cost of mild HAV infection as the sum of the annual treatment costs consisting of direct medical and nonmedical costs as well as productivity losses resulting from lost workdays.

Finally, we conducted sensitivity analyses to determine which parameters exert the greatest influence on the results of this analysis and establish the range of total annual cost of mild HAV infection. A *p*-value ≤ 0.05 was considered to be statistically significant. Epi Info software [25] was used for data entry. Data from questionnaire forms were entered twice into a computer database to reduce the chances of data-entry errors and were analyzed using SAS statistical programs [26].

## Results

Table 1 presents sociodemographic characteristics of the inpatient and outpatient groups. The mean age of the inpatient group was 22 years; the mean age of the outpatient group was 12 years. The inpatient group reported a higher annual household income of US$898.2 (2001 US$) compared with US$502.3 for the outpatient group. Several other comparisons reflected disparities in age of the two sample groups: 26.5% of the inpatient group was employed, compared with 6.7% of the outpatient group; 21% of the inpatient group were married, compared with 8.9% of the outpatient group; 32% of the inpatient group smoked cigarettes, compared with 9.9% of the outpatient group. The average wage rate per patient in urban areas was 14,617 KZT (US$98.76) per month compared to 5000 KZT (US$33.78) per patient in rural areas. There were 27.21 lost workdays for employed inpatients and 6.19 lost workdays for family members caring for minor patients in hospital; employed outpatients had an average of 15.73 lost workdays (lost workdays: 33.40 vs. 15.73). The average length of hospital stay for a patient with mild HAV infection was 9.7 days, compared with 3.4 visits per patient in the outpatient group.

**Table 1:** Sociodemographic characteristics of patients with mild HAV infection, Almaty, Kazakhstan, 2001

Variables	All patients (n = 451)	Inpatients (n = 200)	Outpatients (n = 251)
Gender (%)			
Male	50.9	50.0	51.4
Female	49.1	50.0	48.6
Marital status (%)			
Married	17.8	21.0	8.9
Unmarried	85.2	79.0	91.1
Education level (%)			
Never attended or kindergarten	13.2	0.5	23.4
School 1–8 years	55.8	72.0	42.7
Grades 9–11 years	16.3	27.5	17.3
High school graduate	1.1	-	2.0
College 1–3 years or graduate	13.6	-	14.5
Ethnicity (%)			
Kazakhs	40.6	36.0	44.2
Russians	49.2	54.5	45.0
Uzbek	2.4	2.0	2.8
Others	7.8	7.5	8.0
Employment status (%)			
Employed	16.4	26.5	6.7
Unemployed	83.6	73.5	93.3
Tobacco use (%)			
Smoker	20.3	32.0	9.9
Nonsmoker	79.7	68.0	90.1
Urban status (%)			
Urban	99.8	100.0	99.6
Rural	0.2	-	0.4
Home ownership (%)			
Owned	98.2	99.5	97.2
Rented	1.8	0.5	2.8
Age group (%)			
≤ 18	66.3	37.5	89.2
19 – 26	22.6	42.5	6.8
27 – 34	6.7	12.5	2.0
≥ 35	4.4	7.5	2.0
Mean Age (years) (SE) ^¶^	17 (0.41)	22 (0.49)	12 (0.43)
Mean household income (US$) (SE)^¶^	886.6 (60.03)	898.2 (61.65)	502.3 (28.38)

^¶ ^Standard error (SE) are values in parenthesis.

Table 2 presents the mean treatment costs per patient with mild HAV infection in inpatient and outpatient settings. The mean total direct medical and nonmedical cost of managing mild HAV infection was estimated at US$157.4 (2001 US$) per inpatient (95% CI = US$151 – 163), compared with US$22.5 per outpatient (95% CI = US$21 – 23). The hospital costs (US$81) were calculated by taking the product of the average number of hospital days (9.7 days) and the cost per HAV patient-hospital day (US$8.4) obtained from the survey. Of the US$81 of hospital costs, as much as 40% (US$32) of the hospital costs was allocated to the costs for the purchase and repair of medical equipment and buildings and 60% (US$49) was spent on recurrent hospital administrative and operational costs including overhead and utilities. Laboratory costs accounted for 6.9% (US$11.0) of the mean treatment costs for inpatients compared with 35.6% (US$8.0) of those costs for outpatients. We estimated that about 19% (US$30) of the mean treatment costs were allocated to hospital meals.

**Table 2:** Mean treatment costs of managing cases of mild HAV infection for inpatient and outpatient settings, Almaty, Kazakhstan (2001 US$)

	Inpatient			Outpatient		
		
Cost items (US$)	Mean	SE^†^	95% CI^‡^	Mean	SE^†^	95% CI^‡^
Hospital cost	81.0	1.78	(89.0, 96.0)	-	-	-
Physicians' time	18.0	0.49	(17.0, 19.0)	3.0	0.08	(2.80, 3.20)
Nurses' time	9.0	0.17	(8.7, 9.30)	1.6	0.07	(1.46, 1.70)
Non-health staff	2.0	0.04	(1.92, 2.10)	-	-	-
Laboratory	11.0	0.35	(10.0, 12.0)	8.0	0.11	(7.8, 8.2)
Prescription drugs	6.4	0.21	(6.19, 7.01)	5.9	0.33	(5.25, 6.55)
Hospital meals	30.0	0.57	(29.0, 31.0)	-	-	-
Transport	-	-	-	4.0	0.20	(3.60, 4.40)
Total inpatient/outpatient	157.4	3.04	(151.0, 163.0)	22.5	0.42	(21.0, 23.0)
Productivity losses^¶^	150.9	2.12	(146.7,155.1)	71.5	4.75	(66.8, 76.3)
Total societal cost^§^	308.3	4.90	(298.7, 317.9)	94.0	4.28	(85.6, 102.4)

Note: Data may add exactly due to rounding

^†^Standard error (SE)

^‡^95% Confidence interval (95% CI)

^¶^Productivity losses calculated based on the number of lost workdays reported and average urban wage rate.

^§ ^Total societal costs include treatment costs and productivity losses

From the perspective of the Ministry of Health, the mean direct medical costs of managing mild HAV infection was estimated at US$127.4 per patient in hospital, compared with US$18.5 in an outpatient setting. From the societal perspective, the mean total cost of direct medical and nonmedical services as well as of productivity losses was estimated at US$308.3 per patient in hospital compared with US$94.0 per patient in an outpatient setting. Of the US$157.4 total for direct medical and nonmedical services, a patient in hospital paid approximately US$36.4 out of pocket costs for drugs and hospital meals, compared with US$9.9 for such costs paid by a patient in an outpatient setting.

**Figure 1:**
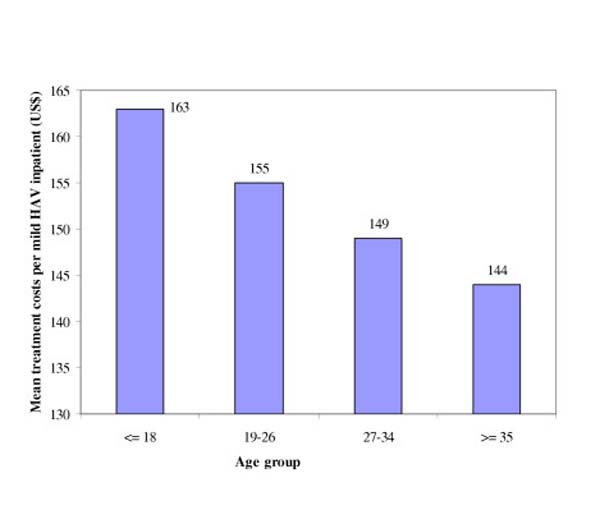
Mean treatment costs per mild HAV inpatient by age group

Results from the PROC TTEST indicated that the difference between the mean treatment costs for management of mild HAV infection among the inpatient and outpatient groups was US$134.6 (2001 US$), 95% CI = (US$ 128.0 – 140.0) and statistically significant at (*p *≤ 0.0001). When we controlled for the effect of age on medical cost by stratifying the samples by age group, we found that the mean treatment costs in the outpatient setting was still considerably lower than those in the inpatient setting. For example, the mean treatment costs of managing cases of mild HAV infection for outpatients aged 19 to 26 years was estimated at 13.9% of that for the inpatients of the same age. The one-way ANOVA analysis indicated that the mean treatment costs per inpatient decreased as age increased (Figure 1). The mean treatment cost per inpatient for the age group ≥ 35 years was estimated at US$144 compared with US$163 for the age group ≤ 18 years (*F*-value = 1.40; *p *= 0.25). Results from this analysis indicated that although there were differences in population means among different age groups, these differences were not statistically significant. We also noted that the mean treatment cost per outpatient in the age group ≥ 35 years was estimated at US$28 versus US$22 for the age group ≤ 18 years (*F*-value = 2.05; *p *= 0.10). Again, results from this analysis indicated that although there were differences in population means, these differences were not statistically significant. Mean treatment costs as well as productivity losses per outpatient increase slightly as age increases (Table 3).

**Table 3:** Mean, median total cost and standard error of managing mild HAV infection, by age group and treatment setting (inpatient vs. outpatient), Almaty, Kazakhstan (2001 US$)

	Total cost* per inpatient			Total cost* per outpatient		
		
Age (years)	n^§^	Mean (SE^†^)	Median cost	n^§^	Mean (SE^†^)	Median cost
≤ 18	75	320 (32.42)	319	224	92 (8.64)	97
19 – 26	85	303 (6.72)	304	17	96 (4.75)	97
27 – 34	25	302 (11.25)	304	5	111 (11.60)	111
≥ 35	15	290 (12.88)	292	5	93 (12.95)	101
All	200	308.3 (4.90)	304	251	94 (4.28)	98

* Note the total cost includes the treatment costs as well as productivity losses

^§ ^Sample size

^†^Standard error (SE)

Nationally, we estimated about 21,600 cases of mild HAV infection occurred during 2001. Of these cases, an estimated 19,008 employed patients with HAV infection would report lost workdays due to illness considering the 12% unemployment rate in Kazakhstan [6]. Assuming no replacement costs of unemployed sick workers, we calculated the productivity losses by multiplying the average lost wages of US$150.9 per inpatient by the cases of employed inpatients. We estimated the productivity losses at US$2.87 million (95% CI = US$2.79 million – US$2.95 million). Using 21,600 cases of mild HAV infection, an average of 9.7 hospital days and 33.4 lost workdays per inpatient, we calculated a total of 209,520 hospital days and 721,440 lost workdays. Annual mild HAV infection treatment costs for inpatients were estimated at US$3.39 million (95% CI = US$3.26 million – US$3.52 million). These costs could be lowered by managing patients with mild HAV infection in an outpatient setting for as low as US$486,000 (95% CI = US$453,600 – US$496,800).

From the societal perspective, the annual total cost of mild HAV infection for patients treated in hospital was estimated at US$6.26 million, 95% CI = (US$6.05 – US$6.47). The treatment costs represent 51% of the total costs whereas productivity losses account for 49% of the total costs. The total cost of managing mild HAV infection in an outpatient was estimated at US$1.85 million, 95% CI= (US$1.72 – US$1.95). The annual total cost of mild HAV infection could be reduced by almost 70% from US$6.26 million to US$1.85 million by encouraging patients with mild HAV infection to be treated in outpatient rather than in inpatient settings. Using an average saving (US$214) per inpatient and 21,600 cases of mild HAV infection, the potential savings at the national level from changing the treatment setting from inpatient to outpatient would be about US$4.62 million (2001 US$) per year.

## Discussion

Our findings suggest that the mean treatment costs per patient managed in hospital were almost seven times the costs per patient in an outpatient setting. Our results were consistent with previous reports that inpatient care is more expensive than outpatient care settings [6]. Even though no other study has directly estimated the costs of mild HAV infection in Kazakhstan, we observed how expensive the inpatient setting in the region was, relative to the outpatient setting. Health systems in the Central Asian Republics, including Kazakhstan, have not been designed and managed with attention to the criteria of *technical efficiency *(the production of a defined set of services at the lowest cost within the health system; also known as operational efficiency) or *allocative efficiency *(the distribution of resources across varied range of services in an optimum manner). These health systems have tended to overinvest services provided in inpatient settings; hospitals utilize approximately 75% of the Kazakhstan health budget compared to 10% of the budget for outpatient care [6]. Many disease entities managed with outpatient treatments in European Union countries (including viral hepatitis and tuberculosis) are managed on an inpatient basis in Kazakhstan [6]. In order to provide optimal care within the budget limits imposed by the allocation of hospital resources to various emerging infectious diseases like human immunodeficiency virus (HIV) infection, health authorities should consider choosing health care practices known to be beneficial on the basis of evidence of cost-effectiveness [27]. Inefficiency in the use of public funds has resulted in the increasing practice of informal or 'under-the-table' payments in the health system [6].

Informal out-of-pocket expenses are increasingly required due to declining funds for the health sector. In the late 1990s, a 65% reduction in government revenues resulted in insufficient health sector funding that led to delays in wage payments and shortage of medical supplies [20]. Although the benefit package in Kazakhstan has covered inpatient drug costs, many hospitals in practice cannot afford to supply them. Therefore, patients often pay for food and drugs while they are in hospital [6]. These expenditures are informal payments that patients are asked to pay out-of-pocket. Our results were consistent with the findings of Ensor and Savelyeva, that estimates of patient per capita payment for prescription drugs in hospital range between US$ 8.5 and US$10.4 [20,28,29]. In Kazakhstan, private practice has been permitted since 1991, and more physicians are becoming semi-autonomous practitioners in group practices funded through a patient capitation fund. Many local health authorities permit health facilities to establish charge departments in order to obtain additional revenue [20]. User charges for medical services by public sector health organizations were legalized in 1995. Now, health care facilities charge for services, and as a result, outpatients make official co-payments for the treatment [6].

In addition to these official charges, recent surveys indicated that informal payments to health care practitioners continue to exist, ranging from US$5 for consultations to thousands for an operation [6,20,30,31]. In this paper, estimates of out-of-pocket expenses were obtained by interviewing patients. Higher costs in inpatient settings may reflect unnecessary use of hospital resources. Other factors that contributed to higher costs in inpatient services may be due to the decrease in government revenues that used to subsidize the hospital services, low official salaries of health care staff, and growth in private health care services [6]. Because of these latter factors, medical practitioners seek augmentation of income via the informal payments [20]. User fees for those who can afford them or cost-sharing strategies could address the problems of under-funding in health systems, stabilize these informal payments, and free up public funds. By balancing the mix of resources and increasing the proportion of spending on services managed in outpatient settings, Ministries of Health in Central Asia might be able to achieve optimal allocation of resources in the health care system.

Our findings may be conservative for three reasons. First, in estimating mean treatment costs, we did not consider informal payments to health care staff. Second, our estimates of productivity losses were based on 264 working days per year (22 working days per month) and did not include the value of housekeeping productivity losses. Third, we used lower estimates of the number of patients with mild HAV as 150 per 100,000; the upper estimates of this figure could be as high as 500 per 100,000.

This study has some limitations. First, we did not evaluate post-treatment health outcomes after hospital discharge, including completion of outpatient visits. However, there is a wealth of literature that has demonstrated a lack of long-term health outcome differences between persons restricted to bedrest or institutionalized and persons forced to exercise or allowed to engage in activities *ad libitum *when symptomatic with acute HAV infection [32,33]. Second, because of lack of reliable data, we were unable to obtain the capital costs of outpatient facilities including the cost of space, maintenance for facility and medical equipment, overhead, and utilities associated with the treatment of mild HAV infection; therefore, our cost estimates for the outpatient might be biased toward the lower end. Despite these potential concerns, these limitations would not invalidate the findings of this study.

In sensitivity analyses, we varied some parameters to establish the range of annual cost estimates of mild HAV infection. We observed that annual cost estimates are sensitive to the following four variables: value of medical services, number of lost workdays, average wage rates, and incidence of HAV infection cases. In these analyses, the annual cost estimates of mild HAV infection varied from US$4.37 to US$24.66 million (Table 4). Considering patients with multiple concurrent diseases or patients diagnosed with severe HAV, cost of medical services can vary significantly.

**Table 4:** Total annual cost estimates of mild HAV infection for inpatients in sensitivity analysis, Kazakhstan, (2001 US$) in millions

Analysis	Treatment cost	Productivity losses	Total cost
Base case assumption	3.39	2.87	6.26
Value of medical services ^†^	4.89	2.87	7.76
Rural wage rate (KZT 5000 per month) ^§^	3.39	0.98	4.37
Greater adjustment of hepatitis A under-reporting^¶^	12.59	12.07	24.66

Note the total cost includes the treatment costs as well as productivity losses

^†^an estimated informal payment is included. When 80–90% informal payment is taken into account, the treatment costs were estimated at US$226.2 per inpatient.

^§ ^reported wage rate based on the survey results

^¶ ^annual incidence of hepatitis A as high as 500 per 100,000 (reported in the Central Asia Republics in 1998)

Future research should consider conducting cost-effectiveness analyses (CEA) taking into account the quality of post-treatment outcomes rather than assuming similar treatment outcomes among inpatients and outpatients. In conducting these analyses, researchers should consider two possible scenarios: (a) the treatment outcome for the inpatient setting is better than that for the outpatient setting (in this case, the inpatient setting is more costly and more effective than the outpatient setting, so an incremental cost-effectiveness analysis should be conducted); and (b) the treatment outcome for the outpatient setting is as good as or better than that for the inpatient setting (thus, because the cost of managing mild HAV infection for outpatients is lower than for inpatients, cost savings should be calculated).

## Conclusion

From a societal perspective, the annual total treatment costs and productivity losses associated with mild HAV infection ranged from US$6.05 million to US$6.47 million for inpatients compared to a range of US$1.72 million to US$1.95 million for patients in the outpatient setting. The results of this study show that compliance with a change in the policy of hospitalization for patients with mild HAV infection could produce savings of US$4.62 million at the national level in Kazakhstan.

## Appendix

Before beginning this study, we obtained institutional review board (IRB) approval (Protocol No. 2708) from the Centers for Disease Control and Prevention (CDC) Assurances-Human Subjects Office. In addition to CDC IRB approval, we also obtained an approval letter from the Ethics Committee Review from the Ministry of Health in Kazakhstan. Prior to administering questionnaires and interviews with patients, we explained the purposes of the study and obtained informed consent forms signed by all adult patients with mild HAV infection. Parents or guardians of all child participants with mild HAV infection under 18 years of age signed the informed consent forms for children. This study was conducted in collaboration with the Data for Decision Making and Policy Branch of the Division of International Health (DIH), Epidemiology Program Office (EPO) of CDC, the CDC Central Asian Regional Office (CDC/CAR), and the Almaty City Sanitary and Epidemiological Stations of the Ministry of Health in Almaty, Kazakhstan.
